# DNA extraction from long-term stored urine

**DOI:** 10.1186/1471-2369-14-238

**Published:** 2013-10-30

**Authors:** Marc Hilhorst, Ruud Theunissen, Henk van Rie, Pieter van Paassen, Jan Willem Cohen Tervaert

**Affiliations:** 1 Clinical & Experimental Immunology, Maastricht University, P.O. Box 5800, 6202 AZ Maastricht, The Netherlands

**Keywords:** DNA, Urine, Vasculitis

## Abstract

**Background:**

Traditionally, for DNA analyses, DNA is recovered from buffy coats. Since DNA in urine has been reported to deteriorate quickly, this option is often not considered. To complete our DNA database in patients with ANCA-associated vasculitis, we aimed to extract DNA from stored urine.

**Methods:**

Urine was stored at the time of kidney biopsy from patients included in our regional kidney biopsy database, who had given informed consent for further study. Urine was subsequently filtered, dialyzed, concentrated and freeze dried and finallyresolubilized and centrifuged. DNA was extracted using the high pure PCR template preparation kit (Roche Diagnostics). Next, concentration and purity were determined by Nanodrop analysis and by Quant-iT analysis.

**Results:**

One hundred and eighty-one patients with ANCA-associated vasculitis were included. Of 114 patients (63%), DNA was available. From 53 of the remaining 67 patients, stored urine was available. Of the 53 samples that were processed, 46 (86.8%) yielded DNA with a mean concentration of 258.7 ng/μL (range 33.2-529) with a mean purity ratio of 1.81 (λ 260/280).

**Conclusion:**

DNA extraction from fresh urine has been described before, yielding DNA usable for PCR analysis in healthy subjects. Storage of fresh urine at 4°C or lower temperatures results in significant degradation of the DNA, making recovery of DNA more difficult with longer periods of storage. In the current study, we demonstrated that DNA could be retrieved from subsequently filtered, dialyzed, concentrated and freeze dried urine that was stored at room temperature. In addition, we demonstrated tthat this DNA could be used for PCR analysis. This method is useful when no other material from these patients is available.

## Background

Genetic factors have been studied by analysis of DNA in many different diseases. In anti-neutrophil cytoplasmic antibody (ANCA)-associated vasculitis, the relevance of this analysis was shown in a genomewide association study [1]. Traditionally, for these analyses, DNA is recovered from buffy coats. Since DNA in urine has been reported to deteriorate quickly [2], urine is generally not used for the purpose of DNA analysis.

We postulate that it is possible to extract DNA from appropriately stored urine from patients with ANCA-associated glomerulonephritis.

## Methods

The Limburg Renal Registry [3] was searched to identify all ANCA positive patients with pauci-immune necrotizing crescentic glomerulonephritis in order to perform DNA analysis. Patients from who no buffy coats and/or tissue was available but who had given consent for further study, were included in the current study. The local Medical Research Ethics Committee of the Maastricht University Medical Centre approved the study.

Exclusion criteria were concomitant renal diseases such as diabetic nephropathy, thin GBM glomerulopathy or anti-GBM glomerulonephritis.

At the time of renal disease, urine of patients was filtered by passing it through a paper filter. The urine was then dialyzed and concentrated in a Proflux M12® (Millipore, Billerica, MA, USA). Dialyzed and concentrated urine was subsequently snap frozen in a bath of liquid nitrogen and dried in a Beta 1–8 LD® freeze dryer (Christ, Osterode, Germany). Samples were subsequently stored at room temperature until use. For this purpose, 0.2 grams of freeze dried urine was resolubilized in 20 mL of MilliQ and left it stirring overnight at 4°C. We next centrifuged the urine samples for 10 minutes at 10000 g. We extracted DNA by using the high pure PCR template preparation kit (Roche Molecular Diagnostics, Mannheim, Germany). Briefly, the supernatant was decanted and the sediment dissolved in 50 μL proteinase K in 1 mL of binding buffer (6 M guaninidine-HCl, 10 mM urea, 10 mM Tris–HCl, 20% Triton X-100, pH 4.4). After a ten minute incubation at 56°C, 100 μL of iso-propanol was added and the solution was centrifuged through a filtertube containing glass fibers for 1 minute at 8000 g. The filtertube was subsequently centrifuged with 500 μL of inhibitor removal buffer (5 M guaninidine-HCl, 20 mM Tris–HCl, 45% ethanol, pH 6.6) for 1 minute at 8000 g and washed three times with wash buffer (20 mM NaCl, 2 mM Tris–HCl, 80% ethanol, pH 7.5) for 1 minute at 8000 g. The DNA on the glass fibers was then eluted in 200 μL of elution buffer by centrifuging for 1 minute at 8000 g and measured for concentration and purity on a NanoDrop® Spectrophotometer (Nanodrop Technologies, Wilmingtom, DE, USA). In addition, DNA was measured using the Quant-iT™ Picogreen® dsDNA assay. This is an ultrasensitive fluorescent nucleic acid staining for quantitating small amounts of double stranded DNA [4].

## Results

In the current study, 181 consecutive patients with ANCA-associated glomerulonephritis were included. Of 114 patients (63%) DNA was available. From 53 of the remaining 67 patients, 24 hour freeze dried urine was available.

**Figure 1:**
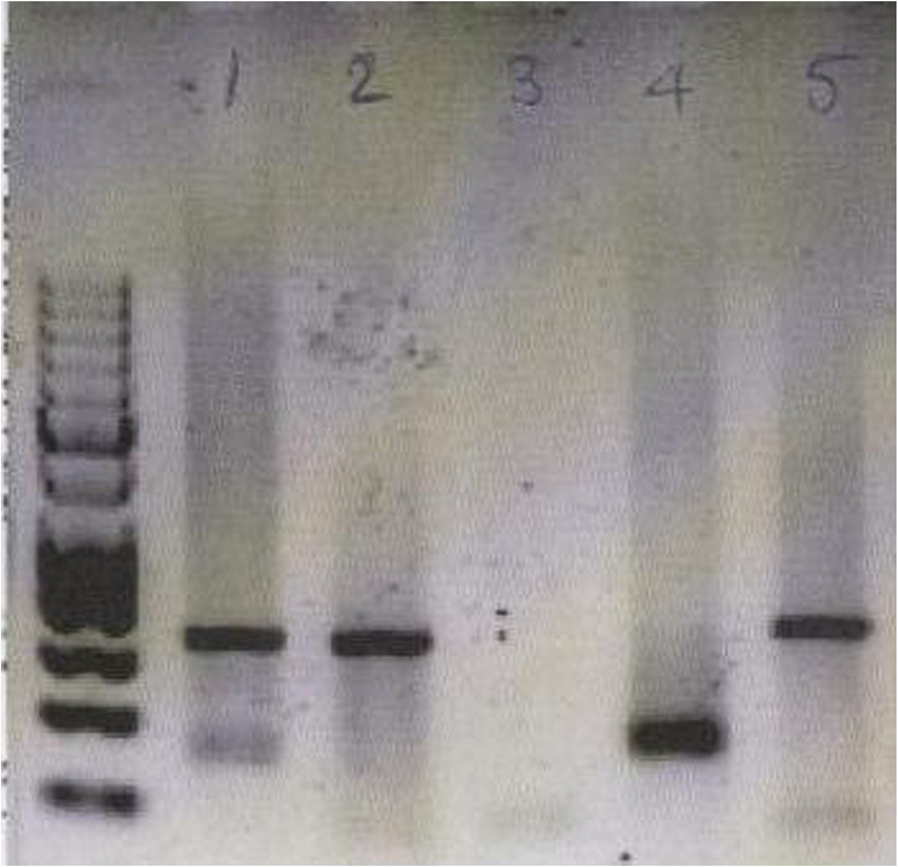
**CTLA-4 +49 polymorphism analyses from 5 patients on DNA retrieved from urine.** Patient 1 had the AG genotype, patient 2 AA, patient 3 non-detectable, patient 4 GG and patient 5 AA.

**Figure 2:**
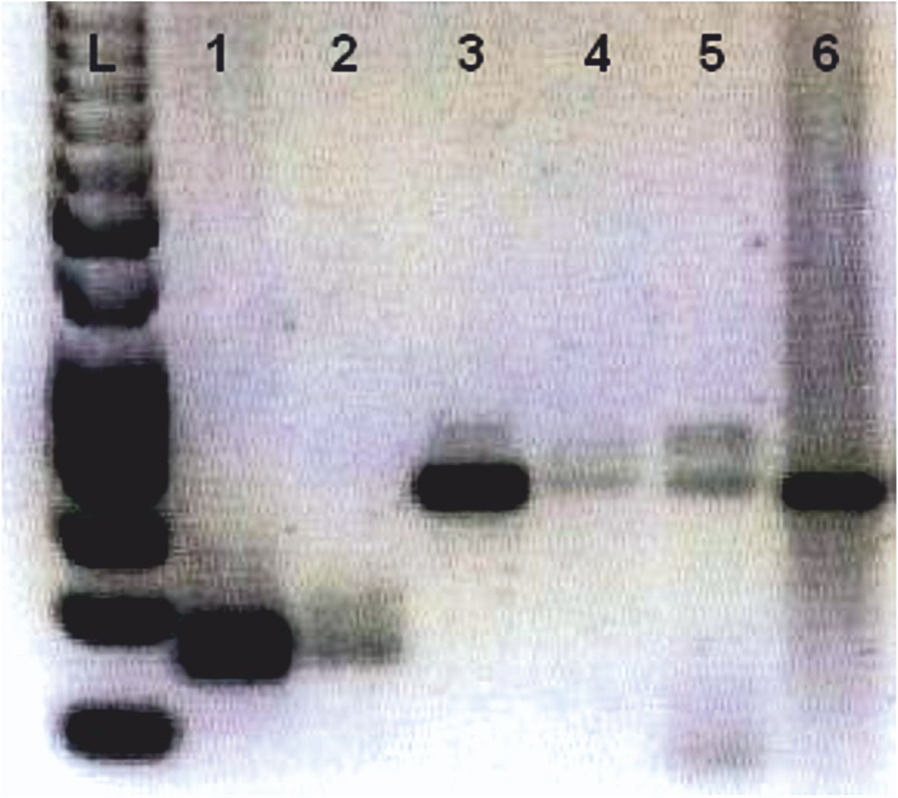
**DNA from urine and DNA from buffycoat from three patients.** The agarose gel shows CTLA-4 +49 polymorphism signals from these three patients. Patient 1 with buffycoat DNA on 1 and urine DNA on 2 (GG); patient 2 with buffycoat DNA on 3 and urine DNA on 4 (AA); patient 3 with buffycoat DNA on 5 and urine DNA on 6 (AA). DNA from urine and DNA from buffycoat yield similar signals in varying magnitude.

Freeze dried urine from these patients had been stored at room temperature for an average time of 16 years (range 6–28). Of the 53 samples that were processed, 46 (86.8%) yielded DNA with a mean concentration of 258.7 ng/μL (range 33.2-529) with a mean purity ratio of 1.81 (λ 260/280) as measured on a Nanodrop® 2100. Eleven samples were further diluted for picogreen analysis. These samples were found to contain DNA in an average concentration of 40 ng/μL on the Nanodrop® 2100 and an average concentration of dsDNA of 23.35 ng/μL by picogreen analysis. Polymorphisms in several genes (CTLA-4, PD1) could be determined in 38 (82.6%) of these samples [5] (Figure 1). Linear regression showed that the amount of DNA extracted from the urine did not correlate with the amount of proteinuria at the time of urine storage (R^2^ = 0.02; p = 0.34) or the length of time that the urine was stored (R^2^ = 0.08; p = 0.55). Furthermore, Chi-square analysis proved gender to be of no significance (p = 0.8) yielding signals in 87.5% of males and in 54.5% of females. Finally, gel electrophoresis with PCR products from DNA from urine and from buffy coat of three patients showed similar bands in varying magnitude (Figure 2).

## Discussion

DNA extraction from fresh urine has been described before, yielding DNA usable for PCR analysis in up to 35% of healthy males and up to 75% of healthy females [2,6,7]. Storage of fresh urine at 4°C or lower temperatures results in significant degradation of human DNA, resulting in low recovery rates during long-term storage [8-13]. When urine is stored at -20°C, around 75% of the DNA degrades within 28 days [11,14], making a quantitative recovery difficult after this period [9]. A temperature of -80°C improves recovery up to 28 days of storage but increases storage costs [15,16]. Importantly, however, Fernandez-Soto *et al.* reported that it was not possible to recover DNA for PCR analysis from urine samples stored at -80°C after storage of 18 months up to 7 years [15]. Adding sodium azide or EDTA has been reported to improve the recovery of DNA [8,12,17,18]. No studies, however, have reported data on DNA recovery from samples stored at -80°C *with* the addition of azide or EDTA. At all temperatures, however, recovery of human DNA after longer periods of storage seems to be difficult. In the current study, we demonstrated that human DNA could be retrieved from freeze dried urine that could be used for PCR analysis, after an average storage period of 16 years at room temperature. Analysis by eosin and haematoxylin (H&E) staining demonstrated damaged but intact cells with intact nuclei in the urinary sediment. Within these cells, leukocytes were present that stained positive with anti-CD45 (data not shown). The cells are clustered around debris in the sediment suggesting that this debris may have protected the cells from degradation during all these years.

## Conclusion

We conclude that in case of deceased or lost-to-follow-up patients, DNA can be retrieved from successively dialyzed, concentrated, and freeze dried urine that has been stored for up to 28 years.

## Abbreviations

DNA: Deoxyribonucleic acid; ANCA: Anti-neutrophil cytoplasmic antibodies; GBM: Glomerular basement membrane; PCR: Polymerase chain reaction.

## Competing interests

The authors declare that they have no competing interests.

## Authors’ contributions

MH, RT and HvR performed extraction of DNA and critical revision of the manuscript. MH and JWCT contributed to the concept of study, interpretation of data, draft of the manuscript and critical revision of the manuscript. PvP contributed to concept of study, interpretation of data and critical revision of the manuscript. All authors have given final approval of the manuscript to be submitted.

## Pre-publication history

The pre-publication history for this paper can be accessed here:

http://www.biomedcentral.com/1471-2369/14/238/prepub

## References

[B1] Lyons P Rayner T Trivedi S Holle J Watts R Jayne D Genetically Distinct Subsets within ANCA-Associated Vasculitis N Engl J Med 2012 367 214 223 10.1056/NEJMoa1108735 22808956 PMC3773907

[B2] Yokota M Tatsumi N Tsuda I Takubo T Hiyoshi M DNA extraction from urinary sediment J Clin Lab Anal 1998 12 88 91 10.1002/(SICI)1098-2825(1998)12:2<88::AID-JCLA3>3.0.CO;2-F 9524292 PMC6807926

[B3] Hilhorst M Wilde B Van Paassen P Winkens B Van Breda VP Cohen Tervaert JW Improved outcome in anti-neutrophil cytoplasmic antibody (ANCA)-associated glomerulonephritis: a 30-year follow-up study Nephrol Dial Transplant 2013 28 373 379 10.1093/ndt/gfs428 23223225

[B4] Ahn SJ Costa J Emanuel JR PicoGreen Quantitation of DNA: Effective Evaluation of Samples Pre-or Post-PCR Nucl Acids Res 1996 24 2623 2625 10.1093/nar/24.13.2623 8692708 PMC145983

[B5] Slot M Sokolowska M Savelkouls K Janssen R Damoiseaux J Cohen TJ Immunoregulatory gene polymorphisms are associated with ANCA-related vasculitis Clin Immunol 2008 128 39 45 10.1016/j.clim.2008.03.506 18448390

[B6] Brinkmann B Rand S Bajanowski T Forensic identification of urine samples Int J Leg Med 1992 105 59 61 10.1007/BF01371242 1354482

[B7] Botezatu I Serdyuk O Potapova G Shelepov V Alechina R Molyaka Y Genetic analysis of DNA excreted in urine: a new approach for detecting specific genomic DNA sequences from cells dying in an organism Clin Chem 2000 46 1078 1084 10926886

[B8] Vu N Chaturvedi A Canfield D Genotyping for DQA1 and PM loci in urine using PCR-based amplification: effects of sample volume, storage temperature, preservatives, and aging on DNA extraction and typing Forensic Sci Int 1999 102 23 34 10.1016/S0379-0738(99)00034-1 10423850

[B9] Van der Hel O Van der Luijt R de Mesquita Bueno H Van Noord P Slothouber B Roest M Quality and quantity of DNA isolated from frozen urine in population-based research Anal Biochem 2002 304 206 211 10.1006/abio.2002.5634 12009697

[B10] Prinz M Grellner W Schmitt C DNA typing of urine samples following several years of storage Int J Leg Med 1993 106 75 79 10.1007/BF01225044 8217868

[B11] Cannas A Kalunga G Green C Calvo L Katemangwe P Reihter K Implications of storing urinary DNA from different populations for molecular analyses PloS One 2009 4 e6985 10.1371/journal.pone.0006985 19746164 PMC2735781

[B12] Ingersoll J Bythwood T Abdul-Ali D Wingood G Diclemente R Caliendo A Stability of Trichomonas vaginalis DNA in Urine Specimens J Clin Microbiol 2008 46 1628 1630 10.1128/JCM.02486-07 18337391 PMC2395082

[B13] Bryzgunova O Skvortsova T Kolesnikova E Starikov A Rykova E Vlassov V Isolation and Comparative Study of Cell-Free Nucleic Acids from Human Urine Ann N Y Acad Sci 2006 1075 334 340 10.1196/annals.1368.045 17108229

[B14] Morré S Van Valkengoed I De Jong A Boeke A Van Eijk J Meijer C Mailed, home-obtained urine specimens: a reliable screening approach for detecting asymptomatic Chlamydia trachomatis infections J Clin Microbiol 1999 37 976 980 10074512 10.1128/jcm.37.4.976-980.1999 PMC88635

[B15] Fernández-Soto P Velasco Tirado V Carranza Rodriguez C Perez-Arellano J Muro A Long-Term Frozen Storage of Urine Samples: A Trouble to Get PCR Results in Schistosoma spp. DNA Detection? PloS One 2013 8 e61703 10.1371/journal.pone.0061703 23613907 PMC3628586

[B16] Elliott P Peakman T The UK Biobank sample handling and storage protocol for the collection, processing and archiving of human blood and urine Int J Epidemiol 2008 37 234 244 10.1093/ije/dym276 18381398

[B17] Milde A Haas-Rochholz H Kaatsch H Improved DNA typing of human urine by adding EDTA Int J Leg Med 1999 112 209 210 10.1007/s004140050237 10335891

[B18] Saetun P Semangoen T Thongboonkerd V Characterizations of urinary sediments precipitated after freezing and their effects on urinary protein and chemical analyses Am J Physiol Renal Physiol 2009 296 F1346 F1354 10.1152/ajprenal.90736.2008 19339629

